# Effect of NaCl and Na_2_SO_4_ on Low Temperature Corrosion of Vapour- and Pack-Aluminide Coated Single Crystal Turbine Blade Alloys CMSX-4 and RR3010

**DOI:** 10.1007/s11661-023-07099-5

**Published:** 2023-06-06

**Authors:** J. Tjandra, A. Ranjan, A. K. Ackerman, M. Appleton, S. Pedrazzini

**Affiliations:** 1 Department of Materials, Imperial College London, Exhibition Road, London, SW7 2AZ UK; 2 Rolls-Royce Plc, PO Box 31, Derby, DE24 8BJ UK

## Abstract

The current work presents a systematic study of two alloy compositions (RR3010 and CMSX-4) and two types of coatings: inward grown (pack) and outward grown (vapour) deposited aluminides, exposed to 98Na_2_SO_4_–2NaCl mixture. Grit blasting was used on some of the samples, prior to coating, to mimic in-service procedures and remove oxides from the surface prior to coating. Two-point bend tests were then performed on the coated samples, with and without applied salt at 550 °C for 100 hours. Samples were pre-strained at 0.6 pct strain to deliberately pre-crack the coating and then strained at 0.3 pct for the heat treatment. Exposure to 98Na_2_SO_4_–2NaCl under applied stress of vapour-aluminide coated samples of both alloys, revealed significant coating damage in the form of secondary cracks in the intermetallic-rich inter-diffusion zone, although only CMSX-4 exhibited cracks propagating further into the bulk alloy while RR3010 proved more resistant. The pack-aluminide coating proved more protective for both alloys, with cracks propagating only into the coating and never into the underlying alloy. In addition, grit blasting proved beneficial in reducing spallation and cracking for both types of coating. The findings were used to propose a mechanism based on thermodynamic reactions, to explain the crack width changes through the formation of volatile AlCl_3_ in the cracks.

## Introduction

Due to the extremely demanding service requirements of turbine blades, significant international effort has gone on for decades into the development of new generations of single crystal, creep resistant, nickel-based superalloys.^[1]^ Nickel-based superalloys are broadly categorised into “generations”, based on increasing Re, Ru and other refractory metal content, usually added at the expense of Cr, Ni, Ti or Co, to improve high temperature creep and fatigue resistance.^[2,3]^ However, the increase the creep strength has come at the expense corrosion resistance, as the content of Cr and Ni was decreased.^[3]^

CMSX-4 is an extremely versatile 2nd generation alloy, widely used to this day in turbine blade applications. It contains only 3 wt pct Re, but has comparatively higher Cr (6.5 wt pct) and Ti (1 wt pct) content when compared to the newer 3rd generation alloys.^[3]^ More creep resistant 3rd generation alloys such as RR3010, contain more than double the Re content (6.8 wt pct), which is added at the expense of Cr (1.7 wt pct) and Ti (0.1 wt pct).^[4]^ Re is an alloying addition known to improve high temperature creep and thermal–mechanical fatigue resistance,^[5]^ and its effect on corrosion has been reported as either beneficial^[6]^ or detrimental^[7]^ in various studies, depending on the exposure and loading conditions. The reduction in titanium content is a welcome change in the new generations of superalloys, as it has been shown to have a strong detrimental effect on oxide scale growth.^[8]^ However, Cr is very commonly added to superalloys to confer corrosion resistance, as it forms a coherent layer of Cr_2_O_3_ on the surface, passivating it and preventing further corrosion.^[9]^ The constant reduction in Cr content with the advancing of new generations of single crystal superalloys has led to the surface oxide scale to form a combination of NiO–Al_2_O_3_ instead of consisting mainly of Cr_2_O_3_.^[10]^ NiO requires higher partial pressures of oxygen to form than Cr_2_O_3_.^[11]^ Al_2_O_3_ is widely considered the more desirable, protective, slow-growing scale, therefore its absence has led to the requirement for improved coatings, to confer some of the oxidation and corrosion resistance that was (necessarily) lost due to the requirement for enhanced creep resistance.^[12]^

Aluminide coatings have been used for decades, sometimes with modifications using Cr, Pt or Si.^[13]^ Most aero-engine manufacturing companies have their own proprietary recipe for the composition and application of their coatings. Nevertheless, coatings play an important role in the oxidation, and therefore the corrosion resistance, of their substrate.^[14,15]^ Coatings can vary from low-cost diffusion-based processes, such as inward grown (pack)-aluminide coating, originally developed in the 1960 to 1970s,^[12]^ from here onwards referred to as “pack aluminide”. Pack aluminide coatings rely on the nickel superalloy being heat treated to create inwards diffusion through the surface layer and therefore an “inward” growth of *β*-NiAl, capable of forming a stable, slow-growing, continuous Al_2_O_3_ scale. Outward growing (vapour) aluminide coatings can be achieved through Chemical Vapour Deposition (CVD), which are from here onwards referred to as “vapour aluminide” coatings.^[12]^

The presence of salt contaminants like NaCl, Na_2_SO_4_ in aero-engines has been shown to accelerate the corrosion process.^[16]^ Molten deposits of such oxides diffuse into the alloy surface and damage both the protective oxides and the underlying alloy, leading to premature failure.^[17]^ In the case of aero-engines, sulfates can be either ingested or formed due to the combustion of sulfur-containing fuel.^[18]^

Corrosion of nickel-based superalloys is broadly categorised as “type-1” high temperature hot corrosion (HTHC) and “type-2” low(er) temperature hot corrosion, where this distinction arises from a change in mechanism. Type-1 Hot Corrosion occurs due to the condensation of fused salts onto the surface of the alloy.^[18]^ This typically happens at the temperature ranges above 884 °C, which is the melting point of sodium sulfate (Na_2_SO_4_). Na_2_SO_4_ can have a fluxing effect on the oxide scale of the nickel superalloy, generally composed of NiO and Al_2_O_3_,^[19]^ thereby reducing its protective effect. Type-2 corrosion is seen within the temperature range 650 °C to 800 °C: it involves the formation of molten salts due to low-melting point eutectics, in mixtures such as Na_2_SO_4_–MSO_4_ (where M can be either nickel or cobalt).^[18]^ Recently, a new mechanism of corrosion was reported at even lower temperatures, between 450 °C and 550 °C^[20,21]^ below the formation temperature of molten eutectic Na_2_SO_4_–MSO_4_.

The objective of our current study is to perform a systematic analysis that will inform the understanding of 4 key variables individually, on low-temperature corrosion resistance:

1. the effect of salt (98Na_2_SO_4_–2NaCl),
2. the effect of applied thermal–mechanical stress (2-point bend testing at ~ 550 °C, to test the effect of the newly reported lower temperature corrosion mechanism),
3. the effect of different coatings—pack (inward grown) or vapour (outward grown) aluminides, grit blasted or not,
4. the effect of the alloy composition (RR3010 or CMSX-4).

RR3010 and CMSX-4 were selected as representative of a 2nd generation and a 3rd generation superalloy, to attempt to elucidate the mechanisms of salt corrosion under applied stress at lower temperatures. Thermal salt exposures were performed using a combination of 98Na_2_SO_4_–2NaCl, which was applied to the surface of coated samples, that were then 2-point bend tested in a furnace, at 550 °C for 100 hours. We coupled characterisation techniques with thermodynamic predictions to propose a corrosion mechanism, with and without applied stress.

## Experimental Methods

In this section we outline the sample composition and experimental parameters used in the current study.

### Samples

RR3010 is a 3rd generation and CMSX-4 is a 2nd generation nickel-based superalloy, both with single-crystal, dual-phase microstructures consisting of *γ*′ precipitate embedded in a *γ* matrix. The nominal chemical composition of RR3010 and CMSX-4 is shown in Table I.

**Table I:** Nominal Chemical Composition of Alloys CMSX-4 and RR3010 in Weight Percent

	Cr	Co	W	Re	Al	Ti	Mo	Ta	Hf	Ni
RR3010	1.7	3.1	5.5	6.8	5.9	0.1	0.5	8.5	—	balance
CMSX-4	6.5	9	6	3	5.6	1	0.6	6.5	0.1	balance

CMSX-4 and RR3010 samples were supplied by Rolls-Royce plc. Both CMSX-4 and RR3010 alloys were produced as cast, homogenised, heat-treated cylinders. Rectangular prisms measuring 55 mm × 3.5 mm × 3.5 mm were cut by electrical discharged machining (EDM) in preparation for the 2-point bend testing experiments.

### Coating

After cutting with EDM, samples were ground using 1200-grit SiC paper to remove any cutting residue prior to surface treatments and the applications of surface coatings. Coating applications were performed at Rolls-Royce plc. Samples were coated using two methods: pack aluminide coating and vapour aluminide coating. Pack aluminide is a diffusion-based technique whereby the coating is grown inwardly from the sample surface using the chemical vapour deposition (CVD) process. In contrast, vapour aluminide coating is a coating that is grown outwardly from the alloy surface, also using the CVD process. Some samples were grit blasted before coating, to assess whether the increased surface roughness would improve coating adhesion.

Temperature and duration of exposure are different depending on the type of coating: pack aluminide coating is performed below 900 °C for ~ 20 hours, while vapour aluminide coating is performed above is 1000 °C for ~ 5 hours. Inward growing coatings are a “pack” powder process (hence the name), in which the metallic component is buried in aluminising powder before heat treatment. Outward growing coatings are processes in which the parts are suspended in a retort or chamber and immersed in a vapour cloud of aluminising gas produced from Al Cr granules. Granules are located on the base of the retort and are covered in Al halide (fluoride or chloride). The halide acts as an activator, facilitiating the generation of an aluminising gas which in turn facilitates deposition on the exposed component surface.

### Salting

Salt solutions were prepared by weighing each constituent (Na_2_SO_4_ and NaCl) using an electronic weighing balance, fixing a desired ratio of 98:2. NaCl used in the present work was supplied by VWR Chemicals with a 100 pct purity. Na_2_SO_4_ was supplied by Sigma Aldrich with a ≥ 99.0 pct purity. Then the saturated aqueous solution was prepared by dissolving the salt mixture in distilled water until the desired concentration. Samples were heated on a hot plate at 100 °C while sprayed with the salt solution. The amount of salt deposited onto the samples was measured by weighing, with a balance accurate to 0.1 mg. The amount of salt deposited per sample was 65 g m^−2^. Only the top surface of the sample (55 mm × 3.5 mm) was salted.

**Fig. 1:**
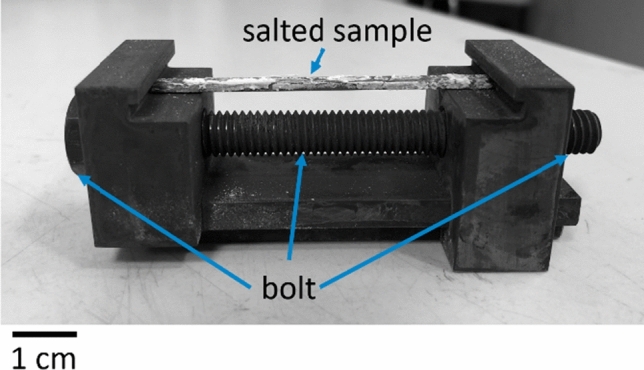
Two-point bend test setup

This method of preparation has been used in previous nickel superalloy corrosion studies because it leads to a randomly inhomogeneous distribution of salt on the surface, therefore it is considered more representative of service conditions.^[17,18,22]^ An example of salted surface is visible in Figure 1. The inhomogeneity and amount of salt deposited is not sufficient to form a coherent salt crust that would inhibit access to air.

### 2-Point Bend Testing

The samples were strained before heat treatment by means of 2-point bend testing, with the full test matrix shown in Table II.

**Table II:** Two-Point Bend Test Experiment Matrix

Alloy	Heat Treated	NaCl-Na_2_SO_4_ Salted	Surface treatment			Heat Treatment Time (h) at 550 °C
			Grit Blasted	Vapour Aluminised	Pack Aluminised	
CMSX-4				√		N/A
	√	√				100
	√			√		124*
	√		√	√		124*
	√				√	124*
	√	√		√		100
	√	√	√	√		100
	√	√			√	100
RR3010	√	√		√		100
	√			√		100
	√			√		100
	√	√		√		100
	√				√	100
	√				√	100
	√	√			√	100
	√	√			√	100

*Some samples were heat treated for longer due to covid-19 pandemic-related lab access restrictions.

2-point bend testing experiments were performed on coated samples, pre-loaded at 0.6 pct strain to crack the coating (the estimated ductility of the coating was 0.5 pct), then unloaded to 0.3 pct strain for the heat treatment at 550 °C. 2-point bend testing was performed with Inconel 718 testing jigs (according to ASTM-G41, using a similar method as previously published literature^[23]^) shown in Figure 1, which were machined in-house and specifically produced for these experiments.

**Fig. 2:**
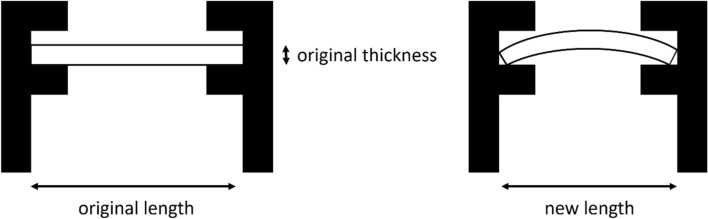
Schematic of two-point bend test. Modifying length is done by adjusting the bolt

To adjust the 2-point bend test jig to the appropriate level of strain, Eq. [1] was used (according to the schematics shown in Figure 2):

1 $${\text{New }}\,{\text{length, }}\,H = \frac{Kt}{\varepsilon }\sin \left( {\frac{L\varepsilon }{{Kt}}} \right)$$

where *K* is an ASTM standard^[24]^ empirical constant equal to 1.28, *L* and *t* are the original length and thickness of the sample respectively and *ε* is the amount of strain. For all length measurements, Vernier callipers were used.

After loading the sample with desired strain (initially 0.6 pct, then 0.3 pct), salting with Na_2_SO_4_–NaCl, and heat-treating for 100 hours, samples were removed from the furnace and air-cooled. Some repeat tests were done for a selected number of samples to randomly check reproducibility of the results. Results were found to be consistent, therefore repeats were not performed for every test condition. There was particular concern about repeatability of the results on the salted samples, as the salt distribution was deliberately applied in an inhomogeneous manner, to mimic real service exposures. When a sample was tested without repeats, multiple cracks were onserved in the same sample, to check consistent behaviour throughout.

### Metallographic Sample Preparation

2-point bend testing samples were mounted sideways with the aid of steel clips, perpendicular to the salted, stressed surface, using Demotec 70 cold-mounting conductive epoxy resin (is a mixture of methyl methacrylate with carbon filler). The resin is thoroughly mixed, poured into the mould and left 24 hours to harden and form an air-tight seal around the sample. Mounted samples were then ground using SiC papers with the following grits: 500, 800, 1200, 2000 and 4000, followed by polishing using 0.04 mm OPS (Oxide Particle Suspension, in this case a colloidal silica suspension) before SEM characterisation. Samples were finally polished with distilled water to remove any OPS residue from the surface.

### Sample Characterisation

The morphology and composition of the oxide scale and corrosion by-products were analysed using scanning electron microscopy (SEM). Samples were analysed using a Zeiss Auriga dual beam SEM–FIB (focussed ion beam) microscope, using secondary electron imaging (topographic contrast) and backscattered electron imaging (Z-contrast), both obtained at 20 kV with a probe size 1 × 10^–8^ A and a working distance of 15 mm. EBSD were acquired using a Zeiss Gemini Sigma3000 field emission gun scanning electron microscope with a Bruker e-Flash^HD^ detector, with a 20 kV accelerating voltage and a pixel size of 0.25 *μ*m. EBSD maps were acquired using the Bruker ESPRIT 3.3 software.

## Results

In this section we present the results of the characterisation and 2-point bend tests of RR3010 and CMSX-4 samples, when exposed to a Na_2_SO_4_–NaCl mixture at 550 °C for 100 to 124 hours.

### Characterisation of As-Cast, Coated Samples

**Fig. 3:**
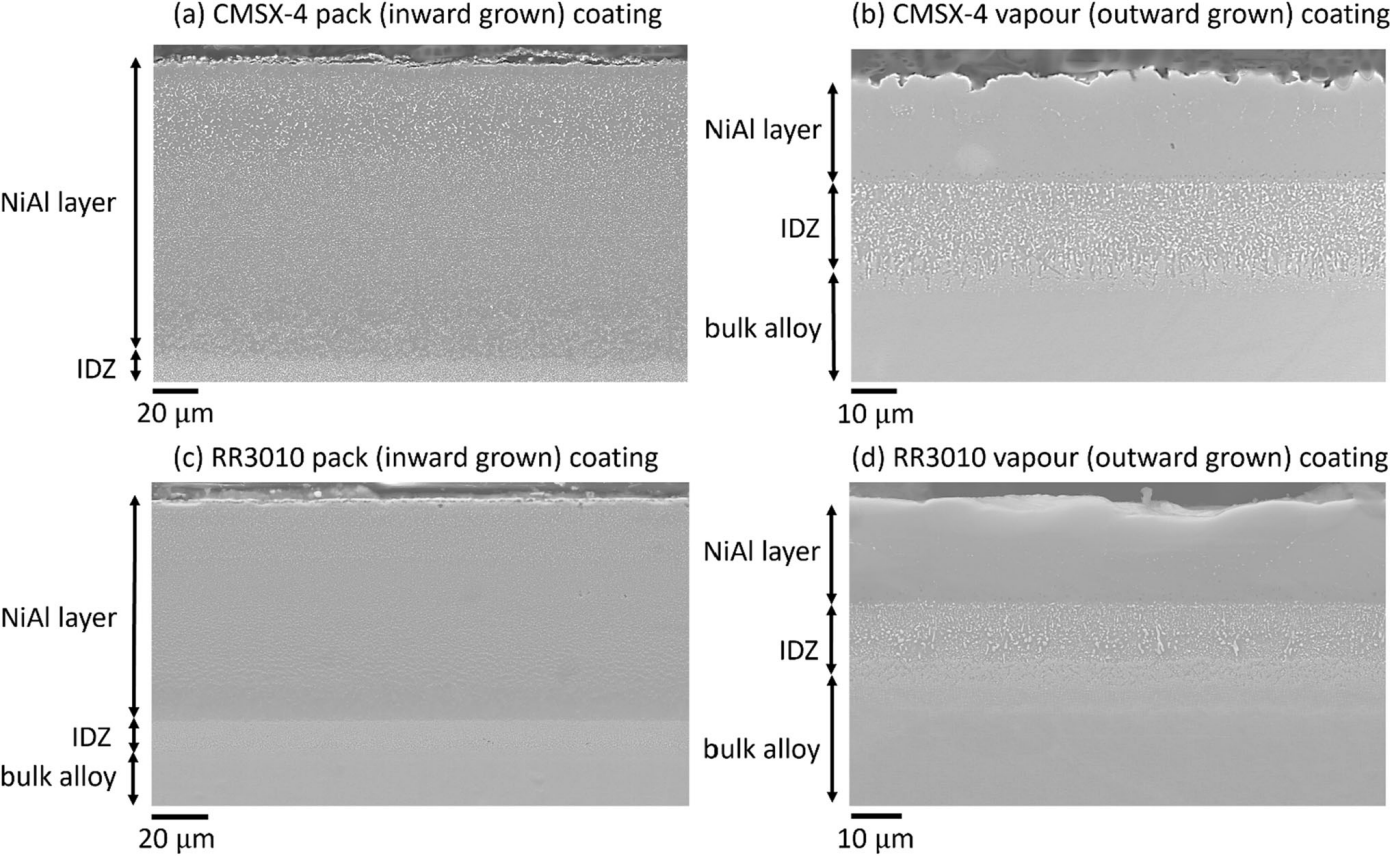
(*a*) Inward grown pack NiAl coating on CMSX-4 sample, (*b*) outward grown vapour NiAl coating on CMSX-4 sample, (*c*) pack aluminide coating on RR3010 sample, and (*d*) vapour aluminide coating on RR3010 sample

CMSX-4 and RR3010 samples were coated using two techniques: pack aluminide and vapour aluminide. The results of the coating procedure were imaged by SEM and are shown in Figure 3.

A cross-section SEM image of CMSX-4 coated with pack *β*-NiAl is shown in Figure 3(a) and RR3010 with the pack *β*-NiAl coating is shown in Figure 3(c). Most of the coating is *β*-NiAl, although as an inwardly grown coating can be inhomogeneous locally, small amounts of residual *γ*-Ni grains remain, as well as TCP phases which formed from residual heavy elements after the NiAl formed. The overall thickness is approximately 80 *μ*m.

A cross-section SEM image of CMSX-4 coated with vapour *β*-NiAl is shown in Figure 3(b) and RR3010 is shown in Figure 3(d). Vapour aluminide coatings were substantially thinner and exhibited more distinct layers, though both coatings seemed to adhere well to the substrate. No porosity or inclusions were observed in the coating and no coating spallation was observed, at this stage.

**Fig. 4:**
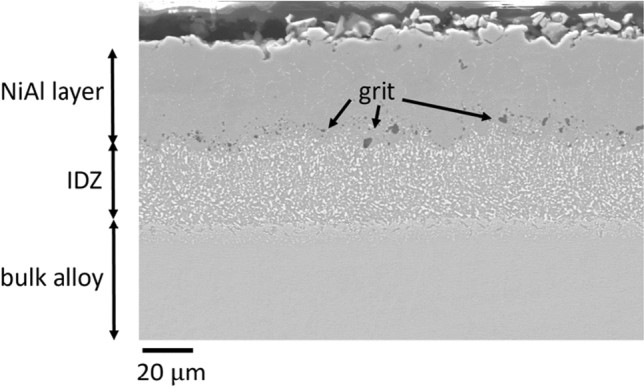
Grit-blasted vapour NiAl coating on CMSX-4

Some of the CMSX-4 samples were grit-blasted prior to the application of the coatings. Grit blasting had the dual purpose of relieving some of the internal stresses present from sample preparation, but also of changing the surface finish to improve coating adhesion. Other studies have found grit blasting to cause recrystallisation of the surface, leading to a reduction in mechanical properties,^[25]^ although this is dependent upon the blasting conditions and any subsequent heat treatments. No evidence of recrystallisation was observed in the present work, as a result of grit blasting, though the surface roughness was visibly altered by the process. Figure 4 shows the more uneven surface finish between the coating and the inter-diffusion zone (visible at the interface between the NiAl precipitation layer and the inter-diffusion zone), with some grit that remained embedded in the samples.

**Fig. 5:**
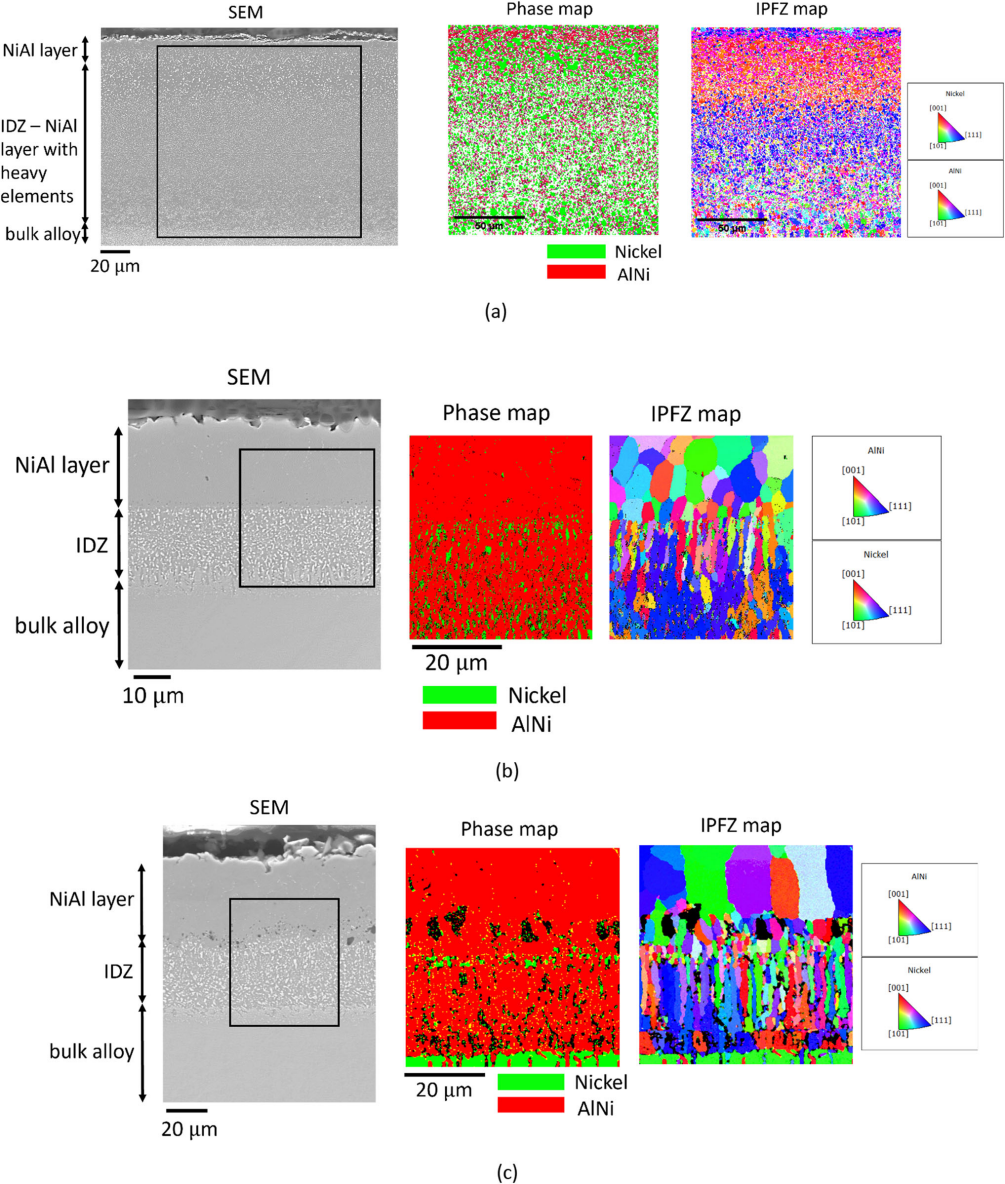
Collage of scanning electron micrographs of each coating type, with their corresponding EBSD phase map and Inverse Pole Figure map (*Z*-axis). (*a*) pack aluminide coated sample, (*b*) vapour aluminide coated sample and (*c*) vapour aluminide coated sample, grit blasted

EBSD was used to gain information on the phases present and the grain size distribution in each coating type: pack-, vapour- and grit-blasted vapour- aluminide, as shown in Figure 5. Figure 5 shows for each alloy, a secondary electron micrograph alongside its corresponding EBSD phase map and inverse pole figure maps taken along the *Z* direction. Figure 5(a) shows that the pack aluminide coated sample contains *β*-NiAl grains, with small amounts of residual *γ*-Ni, and interspersed throughout the whole depth of the sample and inter-diffusion zone. Grain sizes range between 1 and 10 *μ*m, with residual *γ*-nickel grains being on the larger end of the spectrum. The residual TCP phases appear white, as they were largely too small to index at the selected magnification. Figure 5(b) shows the vapour aluminide coating. The *β*-NiAl layer is thicker in this case, compared to the pack aluminide coating shown in Figure 5(a). The phase map shows that both the coating and the inter-diffusion zone are dominated by *β*-NiAl, with small residual nickel grains (2 to 5 *μ*m in diameter). The grain size in the *β*-NiAl coating is generally ~ 10 *μ*m in diameter, with randomly oriented grains. This is a stark contrast with the inwards growing coating, which produced a bigger overall layer, but consisting of smaller grains. Grit blasting increased the grain size in the coating to ~ 20 *μ*m, as shown in Figure 5(c). Individual pieces of grit (SiC) remaining after the grit blasting procedure are visible at the coating-alloy interface in Figure 5(c).

### Mechanical 2-Point Bend Tests of Coated Samples

2-point bend tests were performed in our systematic approach to determine the low-temperature salt corrosion mechanism in nickel superalloys RR3010 and CMSX-4. Previously published experiments determined that 98Na_2_SO_4_–2NaCl has a highly detrimental effect on the corrosion resistance when applied on the alloy surface,^[17]^ in the current work, stress is introduced as an additional factor by means of 2-point bend tests to assess its effect coupled with corrosion. The resulting samples were characterised, and their data are presented in Sections III–B–1 through III–B–3.

#### Pack aluminide coated, 2-point bend tested samples

**Fig. 6:**
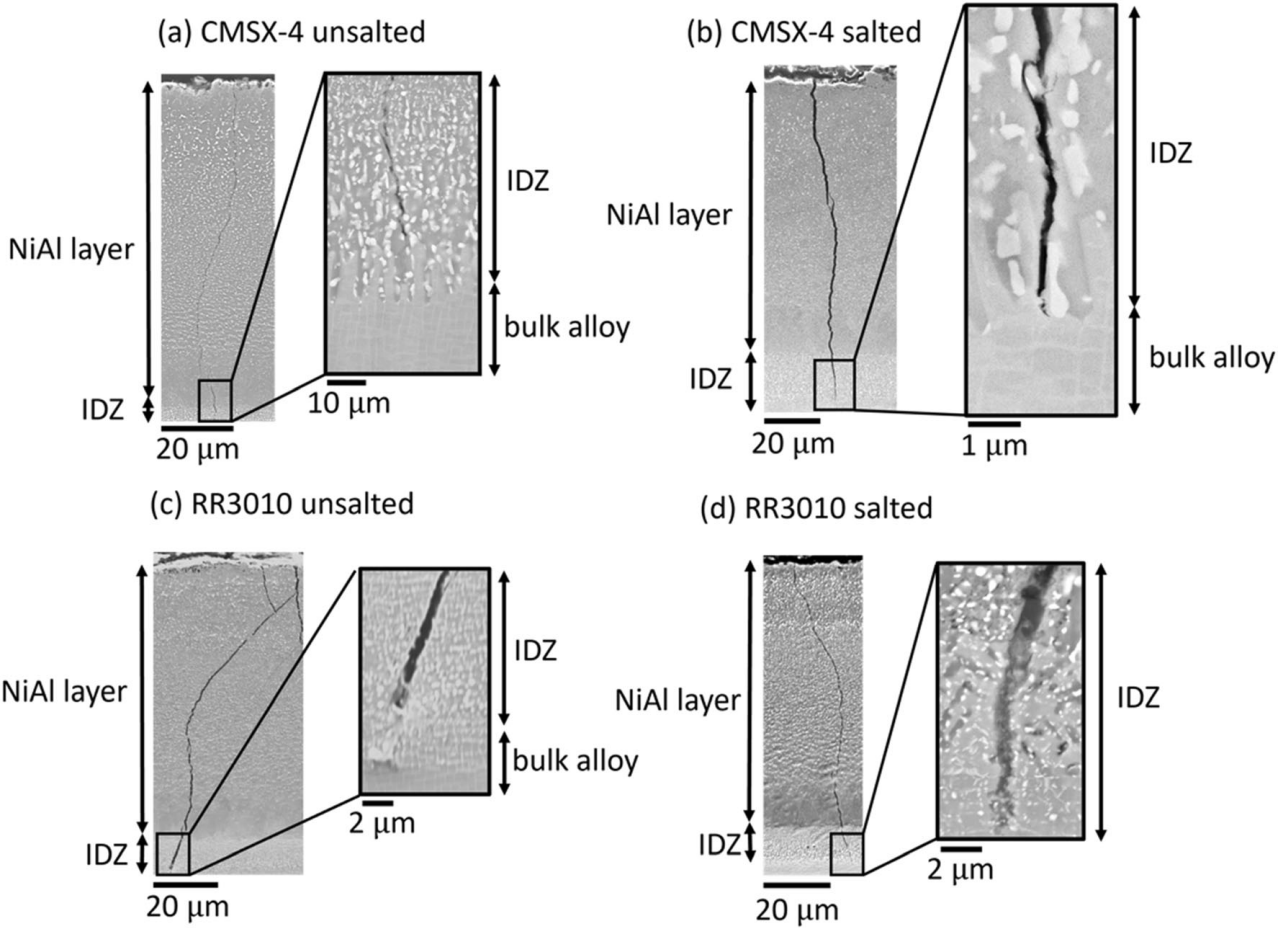
Cross-section image of (*a*) unsalted and (*b*) salted (98Na_2_SO_4_–2NaCl) samples of CMSX-4 tested at 550 °C coated with pack NiAl, with higher magnification at the crack tip. (*c*) unsalted and (*d*) salted RR3010. All samples pre-strained to 0.6 pct, then strained to 0.3 pct during heat treatment

Figure 6 shows cross-sectional imaging of (a) unsalted and (b) 98Na_2_SO_4_–2NaCl salted samples of CMSX-4 coated with pack aluminide coating, as well as (c) unsalted and (d) salted RR3010 coated with pack aluminide coating. Samples were pre-strained to 0.6 pct (~ 660 MPa) to deliberately crack the coating, and heat treated while strained at 0.3 pct (~ 330 MPa) for 100 hours (salted) at 550 °C.

As shown in Figures 6(a) through (d), the cracks in unsalted samples of both CMSX-4 and RR3010 alloys (Figures 6(a) and (c)) propagated all the way through the coating, as intended by the pre-cracking process, but stopped at the coating-substrate interface and did not propagate into the underlying bulk alloy. This is evident from the higher magnification inlays of the crack tips shown in Figures 6(a) and (c), showing that cracks stop at the coating-alloy interface. This is similarly the case for the salted sample of CMSX-4 (Figure 6(b)). However, the salted sample of CMSX-4 had, on average, wider cracks than the unsalted sample, as seen from the lower magnification image in Figure 6(b). In all samples, cracks initially propagated in a relatively straight line perpendicular to the sample surface, however, some crack deflection around precipitates was occasionally observed regardless of salt application. Unlike on CMSX-4, the presence of salt on RR3010 didn’t make the cracks wider, though it increased the average a few microns into the underlying alloy, whereas previously the cracks stopped at the coating-alloy interface. Though initially straight, cracks appeared less linear and more tortuous as they propagated on the coating of the RR3010 samples, compared to the CMSX-4.

**Fig. 7:**
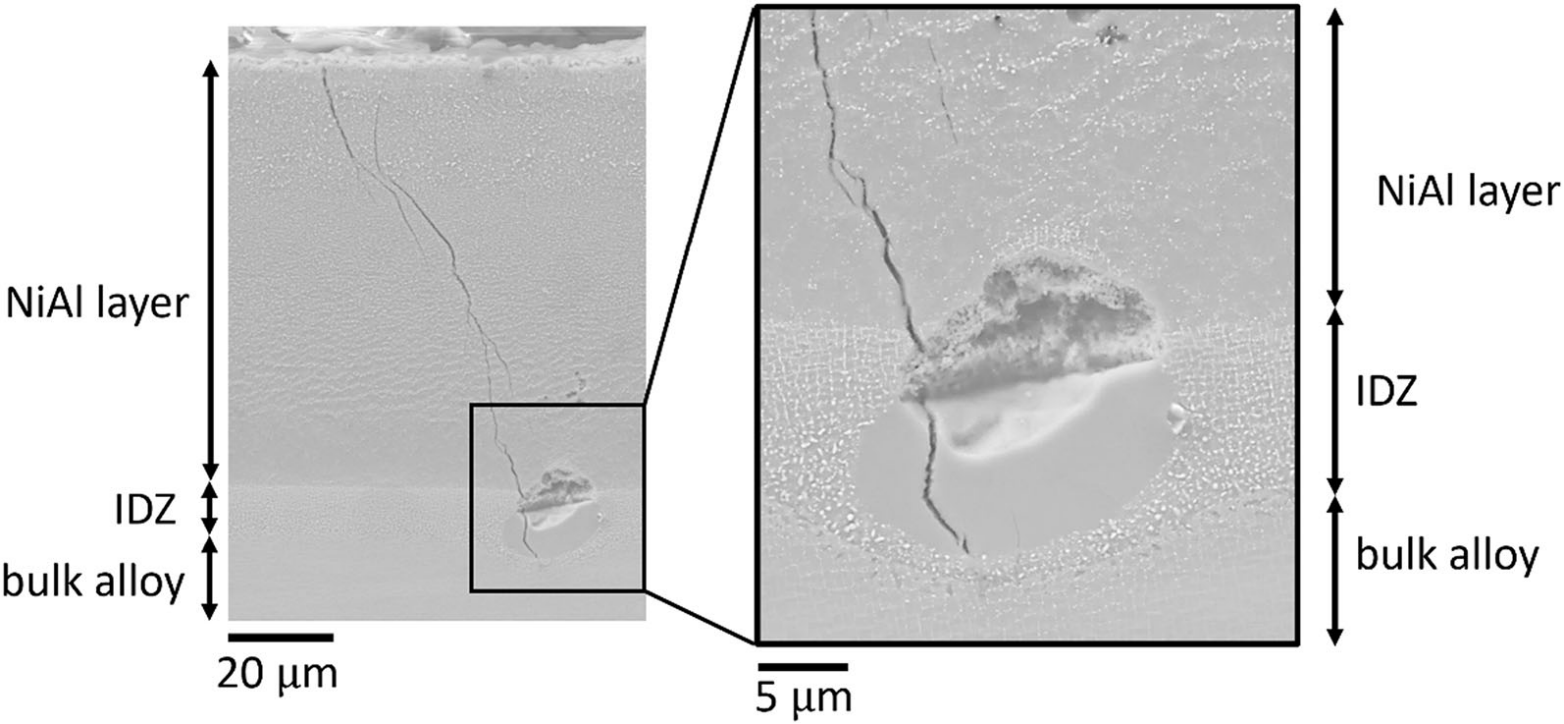
Cross-section image of a salted (98Na_2_SO_4_–2NaCl) sample of RR3010 tested at 550 °C coated with pack NiAl, with higher magnification at the crack tip. Samples pre-strained to 0.6 pct, then strained to 0.3 pct during heat treatment for 100 h (salted) and 124 h (unsalted). A large inclusion is present at the coating- alloy interface

In addition, some oxide inclusion sites were found in the RR3010 pack aluminide coated samples when imaged by SEM. We are showing these inclusions in this manuscript for completeness, although a detailed analysis of their origin and full effect is beyond the scope of the current work. A cross-sectional image of the salted RR3010 sample, coated with the pack aluminide coating, is shown in Figure 7, including a higher magnification inlay of the inclusion at the edge of the inter-diffusion zone. A total of 7 inclusions were found, typically between 5 and 10 *μ*m wide. The inclusions likely created a localised stress concentration, because based on the data collected, the probability of cracks being found at the inclusions sites was more than 50 pct (four out of seven inclusions observed had cracked). These inclusions were only observed in the salted RR3010 sample and not in the CMSX-4 sample.

The width of the inclusion shown in Figure 7 was around 12 *μ*m. The crack formed from the bend tests seems to propagate through this inclusion and towards the end of the inter-diffusion zone, but the crack stopped before entering a region of *γ*/*γ*′ microstructure.

#### Vapour aluminide coated samples

**Fig. 8:**
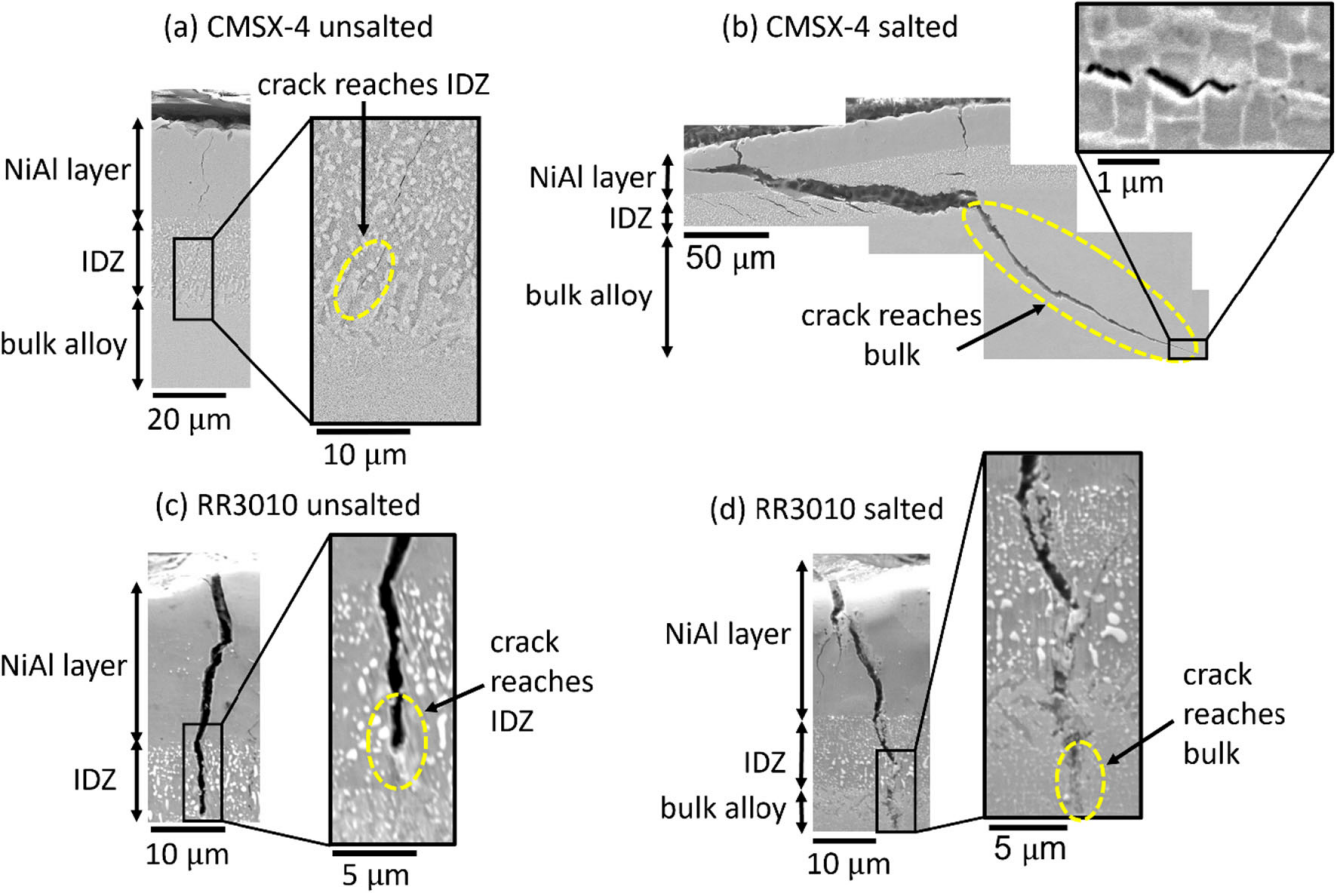
(*a*) Cross-section image of CMSX-4 coated with vapour aluminide, unsalted. (*b*) Cross-section image of salted CMSX-4 coated with vapour aluminide. Crack tip is shown on the right. (*c*) cross section of RR3010 unsalted sample. (*d*) Cross section of RR3010 salted sample

The effect of the addition of 98Na_2_SO_4_–2NaCl on vapour aluminide coated CMSX-4 and RR3010 samples was investigated. This is shown in Figure 8. Figure 8(a) shows the unsalted CMSX-4 sample, (b) shows the salted CMSX-4 sample, (c) shows the unsalted RR3010 sample and (d) shows the salted RR3010 sample.

From Figure 8, the unsalted CMSX-4 vapour aluminide coated sample (Figure 8(a)) has a very thin crack that stops at the coating, much like the unsalted pack-aluminide coated CMSX-4 sample had, in Figure 6(a). However, the salted CMSX-4 sample cracked in a completely different way: the main crack was found in the centre of the sample, which experienced the highest applied stress based on the geometry of 2-point bend tests, and it propagated sideways, at 45 deg throughout the coating and ~ 300 *μ*m into the underlying bulk alloy. Substantial damage of the coating occurred, apart from the large central crack, many secondary cracks appeared at 45 deg in the inter-diffusion zone, as shown in Figure 8(b). Furthermore, secondary cracks only formed in the most brittle part of the coating—the section which exhibits the most intermetallic precipitation—but did not propagate all the way to the outer surface or into the bulk alloy. Furthermore, the coating was partially lifted off the sample surface, which was no longer flat after the 2-point bend test. The crack that propagated into the bulk alloy, did not appear to follow the $\gamma$ channels or any specific microstructural feature.

The salted RR3010 sample (Figure 8(d)) has undergone more damage than its unsalted counterpart (Figure 8(c)), but while the cracking was clearly exacerbated by the presence of salt, cracks reached the *γ*–*γ*′ microstructure but did not propagate within it beyond a few mm. The same thing happened to the salted vapour aluminide coated RR3010, shown in Figure 6(d). In Figure 8(d), multiple cracks can be seen propagating in the intermetallic rich section of the inter-diffusion zone. While only one crack is shown in Figure 8(d), all 4 samples analysed had cracks that extended to the end of the coating and reached the base alloy. Salting also increased the crack width: the average width of cracks in unsalted samples and salted samples was 0.7 and 1.2 *μ*m respectively.

#### Effect of grit blasting

**Fig. 9:**
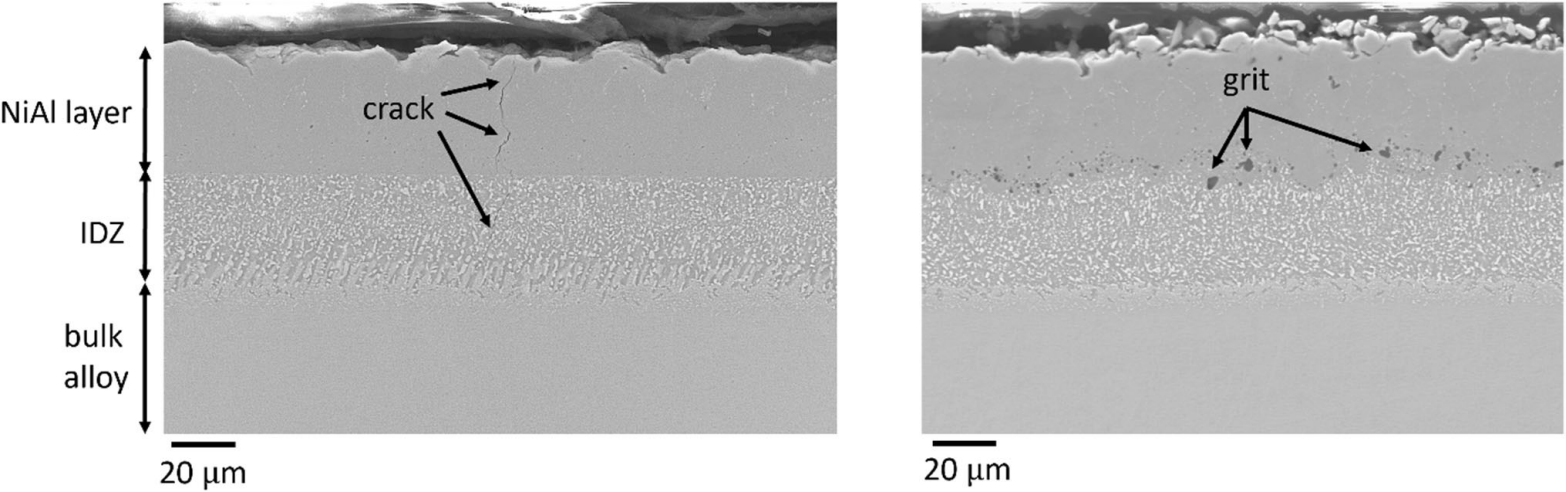
Cross-section image of bare and grit-blasted samples of CMSX-4, both coated with vapour NiAl. Note that no cracks were observed in the grit-blasted sample. Samples pre-strained to 0.6 pct, then strained to 0.3 pct during heat treatment

Some samples of CMSX-4 were grit-blasted prior to being vapour aluminide coated. In the grit blasted samples, coatings did not crack, even when pre-strained to 0.6 pct, while the bare samples had cracked under the same stress conditions, as shown in Figure 9.

**Fig. 10:**
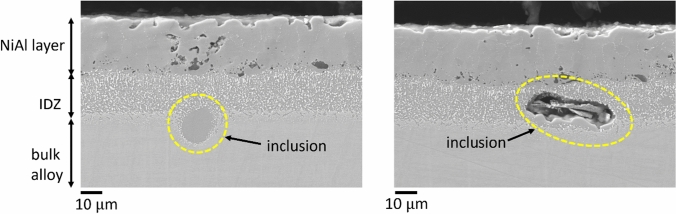
Cross-section image of salted grit-blasted samples of CMSX-4 coated with vapour NiAl. A few inclusions were observed at the coating-alloy interface. Note that one very thin crack, about 30 *μ*m deep, was observed in the salted grit-blasted sample. Samples pre-strained to 0.6 pct, then strained to 0.3 pct during heat treatment at 550 °C

The non-grit blasted coating was shown to have a ductility of 0.5 pct, which is the reason pre-straining of samples prior to heat treatments was done to 0.6 pct strain therefore fracturing the non-grit blasted coating. Grit blasting relieves internal stresses in the coating layer, thereby preventing localized stress from exceeding the yield stress, keeping the coating from cracking. Grit blasting also caused the surface roughness to increase, allowing better adhesion of coating.^[26]^ Furthermore, grit introduces some compressive stresses which prevented the formation of cracks during bend test when the top surface of the sample experiences tensile stresses. This is evident in the salted grit-blasted CMSX-4 coated with vapour NiAl sample, shown in Figure 10. Only one very thin crack was present in the entire sample, which was ~ 30 *μ*m deep. This is in comparison to the salted CMSX-4 coated with vapour NiAl that was not grit-blasted, shown in Figure 8(b), which had very significant damage and cracking through the coating layer into the bulk alloy. The grit-blasted sample did however have some inclusions present in the coating-alloy interface as shown in Figure 10.

Experimental results are summarised in Table III.

**Table III:** Summary of Samples, Coatings, Processing and Experimental Conditions

		CMSX-4			RR3010		
		As-Coated	2-pt Bend Tested		As-Coated	2-pt Bend Tested	
			Air	Air + Salt		Air	Air + Salt
Pack Aluminide	non-grit blasted	uniform ~ 80 *μ*m thick coating constituted primarily of NiAl intermetallics	thin cracks throughout the coating, stopping at the inter-diffusion zone	thicker cracks throughout the coating, stopping at the inter-diffusion zone	uniform ~ 80 *μ*m thick coating constituted primarily of NiAl intermetallics	thin cracks throughout the coating, stopping at the inter-diffusion zone	thin cracks throughout the coating, stopping slightly beyond the inter-diffusion zone
Vapour Aluminide	non-grit blasted	NiAl coating, ~ 25 *μ*m thick	thin cracks throughout the coating, stopping at the inter-diffusion zone	thickest crack, throughout coating and into the bulk alloy (~ 300 *μ*m). Secondary cracks in the inter-diffusion zone	NiAl coating, ~ 25 *μ*m thick	thick cracks throughout the coating, stopping at the inter-diffusion zone	thick, tortuous cracks throughout the coating, stopping slightly beyond the inter-diffusion zone
	grit blasted	NiAl coating, less uniform interface with embedded grit particles. ~ 25 *μ*m thick	did not crack	one very thin crack, 30 *μ*m deep, did not penetrate the entire coating layer	—	—	—

## Discussion

In all the samples that cracked, cracks initiated at the surface. This was established partly from observation, but was consistent with the understanding that in 2-point bend tests, the maximum stress is at the surface. The samples were deliberately pre-cracked prior to the furnace heat treatments and the *evolution* of the cracks under applied heat and environmental factors was assessed. The differences in evolution of the cracks can be due to a combination of the following factors which we discuss in Sections IV–A and IV–B: coating type, including adhesion of the coating to the substrate, thickness and ductility of the coating and environmental factors (salt application, heat treatment).

### Effect of Coating Type

Pack aluminide coating produced an ~ 80 μm thick layer of *β*-NiAl intermetallics with small amounts of residual *γ*-nickel grains and TCP phases interspersed, with a gradual transition to the bulk *γ*–*γ*′ microstructure of the underlying alloy. Vapour aluminide coating, on the other hand, produced a thinner ~ 25 *μ*m coating, with a layer of *β*-NiAl, some TCP phases and residual *γ*-nickel. The transition between the coating and the bulk material was more abrupt in this case, less gradual than the pack aluminide coating. Both coatings exhibited similar levels of ductility, cracking at 0.6 pct applied strain although grit-blasting was shown to prevent cracking and improve ductility. However, the thinner dual-layer vapour aluminide coating proved less protective and more prone to delamination when salt was applied and the coating was deliberately cracked.

Larger grain sizes and a less gradual separation between the coating layer and the bulk alloy of vapour aluminide coated samples were deemed responsible for exacerbating damage under applied stress.^[27]^ Furthermore, the more abrupt transition between the coating and the bulk alloy in vapour aluminide coated samples resulted in weaker bonding between layers, increasing the likelihood of delamination^[28]^ and crack propagation perpendicularly to the coating surface. Subsequently, both the primary and secondary cracks in the intermetallic rich inter-diffusion zone (such as the ones observed in Figure 8) propagated at an angle of about 45 deg, instead of 90 deg, to the coating surface. The primary crack then proceeded at 45 deg in the underlying bulk alloy, which where the maximum critical resolved shear stress is experienced according to Schmid’s law.^[29]^

The protective nature of an intact *β*-NiAl layer was demonstrated by the fact the grit-blasted vapour aluminide coated samples did not crack or exhibit any measurable form of corrosion, even when salt was applied to the surface prior to mechanical testing. However, the cracked *β*-NiAl coating in the non-grit blasted samples lost its protective effect. While grit blasting has been proven to decrease the environmental resistance of some alloys in fatigue tests, particularly after recrystallisation was observed at the surface,^[25]^ our work shows an improvement when pre-loading in 2-point bend conditions.

The presence of inclusions at the coating-alloy interface, observed only in vapour aluminide coated RR3010 alloy, is expected to be a result of some of the reported application drawbacks of RR3010 itself: previous studies reported secondary reaction zone phase instability in the base alloy adjacent to the coatings.^[3]^ These inclusions were only observed in the outward growing vapour aluminide coating, as the inward growing pack aluminide coating exhibited a more gradual, less abrupt transition between the coating and the base alloy.

### Effect of Salt Exposure

Unsalted CMSX-4 samples, with either coating, had extremely thin cracks when compared to all other samples. The addition of salt generally widened pre-existing cracks on the CMSX-4 alloy samples (regardless of coating), which is an indicator of chemical reaction(s) occurring between the coating and the salt. In the case of the vapour aluminide coated sample, salt addition increased both width and depth of the cracks.

In RR3010, on the other hand, crack thickness was related to the type of coating applied: the pack aluminide coating had thin cracks, while the vapour aluminide coating had thicker cracks. Salt application extended the depth of the cracks, but did not visibly alter their width (although they were wider than the CMSX-4 cracks to begin with). Note that the experimental setup involved pre-straining the sample to 0.6 pct to deliberately crack the coating prior to heat treatments.

**Fig. 11:**
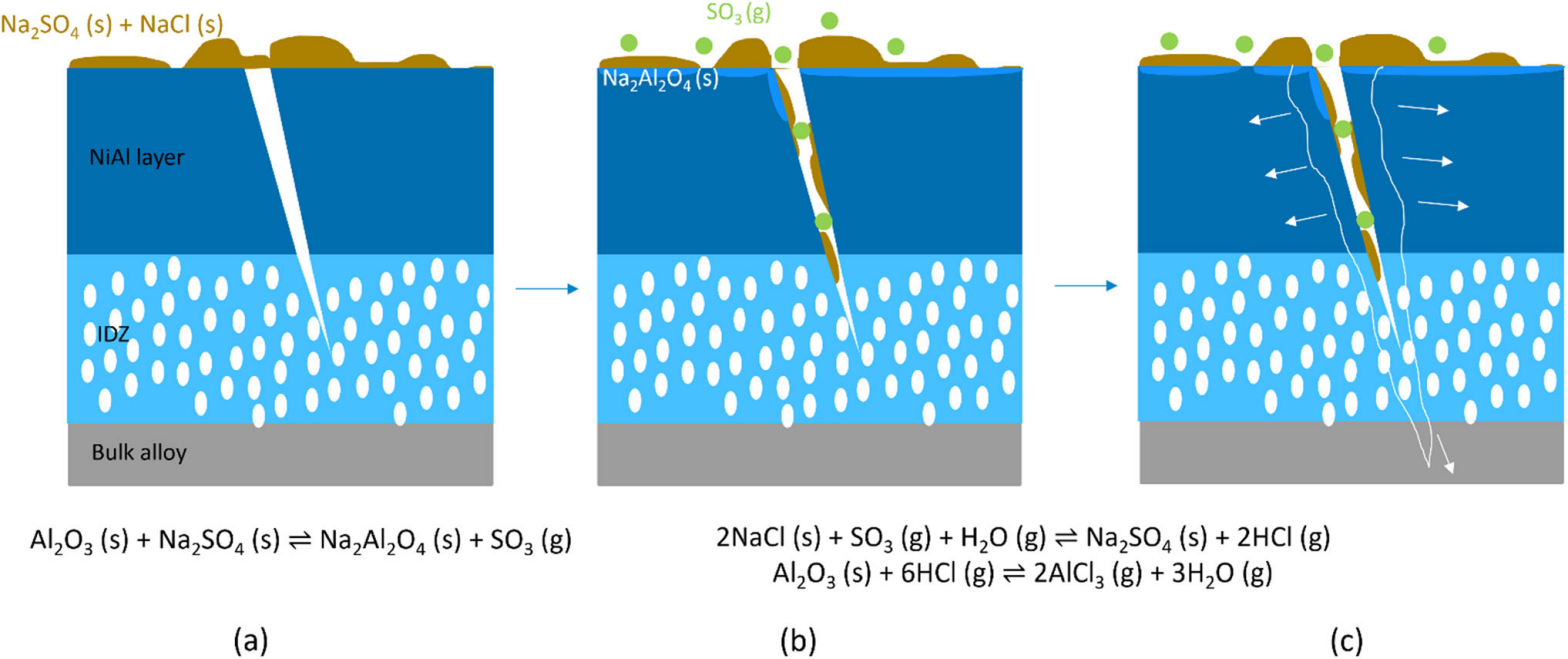
Schematic showing the proposed reaction mechanism observed on vapour aluminide coated samples of RR3010 and CMSX-4 due to the addition of salt. (*a*) the alumina and sodium sulfate combine on the surface to create sodium aluminate and sulfur trioxide, (*b*) the combination of gaseous sulfur trioxide and sodium chloride then releases hydrochloric acid in the presence of moisture in the air, (*c*) the hydrochloric acid then reacts with the remaining alumina turning it into volatile aluminium chloride

A thermodynamic mechanism explaining the increased damage on the vapour aluminide coated samples (of either alloy) is proposed in Figure 11.

The mechanism is proposed as follows. An initial reaction between salt and alumina occurred (Figures 11(a) and (b))^[30]^:

2 $$\begin{array}{ll} {{\text{Na}}_{2} {\text{SO}}_{4} \left( {\text{s}} \right) + {\text{Al}}_{2} {\text{O}}_{3} \left( {\text{s}} \right) \rightleftharpoons {\text{Na}}_{2} {\text{Al}}_{2} {\text{O}}_{4} \left( {\text{s}} \right) + {\text{SO}}_{3} \left( {\text{g}} \right)} \\ \end{array}$$

While Na_2_SO_4_ and Al_2_O_3_ are both stable oxides and salts, Na_2_Al_2_O_4_ can be produced in small quantities. A by-product of this reaction is the localised generation of SO_3(g)_, which can react further with NaCl (this is a similar reaction for the manufacture of Na_2_SO_4_, termed the Hargraeves process^[31]^):

3 $$\begin{array}{ll} {2{\text{NaCl }}\left( {\text{s}} \right) + {\text{SO}}_{3} \left( {\text{g}} \right) + {\text{H}}_{2} {\text{O}} \left( {\text{g}} \right) \rightleftharpoons {\text{Na}}_{2} {\text{SO}}_{4} \left( {\text{s}} \right) + 2{\text{HCl}} \left( {\text{g}} \right)} \\ \end{array}$$

This reaction produces hydrogen chloride gas, which is highly reactive and can the react with Al_2_O_3_ present in the coating layer:

4 $$\begin{array}{ll} {{\text{Al}}_{2} {\text{O}}_{3} \left( {\text{s}} \right) + 6{\text{HCl }}\left( {\text{g}} \right) \rightleftharpoons 2{\text{AlCl}}_{3} \left( {\text{g}} \right) + 3{\text{H}}_{2} {\text{O}} \left( {\text{g}} \right)} \\ \end{array}$$

The reaction involving SO_3(g)_ (Eq. [3]) is exacerbated in the crevice, due to localised increase in SO_3_ partial pressure due to the fact the gas is trapped, driving the reaction further according to Le Chatelier’s principle.^[32]^ The resulting acidification of the crack tip of the scales can be linked to studies done by Rapp.^[19,33,34]^ As Al_2_O_3_ is converted to AlCl_3_, which sublimes at 181 °C, the reaction (Eq. [4]) effectively consumes the alumina from the sample, hence widening and deepening the cracks on the vapour aluminide coated samples. Furthermore, Figure 8 shows significant damage of the coating on the CMSX-4 sample and cracks propagating > 100 *μ*m into the bulk alloy. This is likely due to the reduced tensile strength and creep resistance of alloy CMSX-4 compared to alloy RR3010. This could also be due to the effect of rhenium on the corrosion resistance of both alloys. While both alloys contain substantial amounts of rhenium (3 pct in CMSX-4 and 6.8 pct in RR3010, see full compositions in Table I), rhenium can have a positive effect on the corrosion resistance of nickel-based superalloys.^[6]^

With regards to the pack-aluminide coated samples, the cracks were thicker with the application of salt but not deeper on the CMSX-4 samples, whereas they were deeper but not thicker on the RR3010 samples. The lack of alumina layer on the current samples likely played a role in this observation, as it is actively involved as a reagent in mechanism proposed for the vapour aluminide coated samples. Also—the pack-aluminide coating, by creating more of a diffuse transition to the substrate, likely enhanced adhesion and resulted in all-round better performance in both type of samples.

While most of the corrosion resistance is conferred by the sluminide coatings, there is undeniably an effect of composition on the crack evolution. The crack depth increase on both RR3010 samples (vapour- and pack-aluminide coated) is likely related to the difference in composition when compared to CMSX-4.

## Conclusions

To simulate and understand service conditions, the effect of salts on coated samples under applied stress was assessed. The selected 98Na_2_SO_4_–2NaCl salt mixture was applied on pack- and vapour-aluminised (coated) CMSX-4 and RR3010 samples, at 550 °C. Two-point bend tests were performed with and without the salt mixture at 550 °C for 100 to 124 hours. The two-point bend tests revealed that:

1. The addition of salt caused wider cracks to form in both coatings, on both alloys compared to unsalted samples.
2. Grit blasting prevented cracks from forming by improving the ductility and the adhesion of the coating. However, the presence of inclusions or porosity exacerbated the effects.
3. The effect of salt on pack- aluminide coated samples was relatively minor, on both alloys. Some spallation of the coating surface was observed on the salted sample, which was not present in the unsalted sample. Cracks extended only through the coating and did not penetrate the underlying bulk alloy samples.
4. A significant difference was observed in vapour-aluminide coated samples. The coating was severely damaged when exposed to salt, and cracks propagated beyond the coating, into the bulk CMSX-4 alloy. The RR3010, while undergoing more damage than the unsalted sample, did not have cracks penetrating the bulk alloy.
5. A proposed mechanism involving the formation of volatile AlCl_3_ was used to explain the widening and deepening of cracks under these conditions.
